# The Genome Organization of *Thermotoga maritima* Reflects Its Lifestyle

**DOI:** 10.1371/journal.pgen.1003485

**Published:** 2013-04-25

**Authors:** Haythem Latif, Joshua A. Lerman, Vasiliy A. Portnoy, Yekaterina Tarasova, Harish Nagarajan, Alexandra C. Schrimpe-Rutledge, Richard D. Smith, Joshua N. Adkins, Dae-Hee Lee, Yu Qiu, Karsten Zengler

**Affiliations:** 1 Department of Bioengineering, University of California San Diego, La Jolla, California, United States of America; 2 Pacific Northwest National Laboratory, Richland, Washington, United States of America; Uppsala University, Sweden

## Abstract

The generation of genome-scale data is becoming more routine, yet the subsequent analysis of omics data remains a significant challenge. Here, an approach that integrates multiple omics datasets with bioinformatics tools was developed that produces a detailed annotation of several microbial genomic features. This methodology was used to characterize the genome of *Thermotoga maritima*—a phylogenetically deep-branching, hyperthermophilic bacterium. Experimental data were generated for whole-genome resequencing, transcription start site (TSS) determination, transcriptome profiling, and proteome profiling. These datasets, analyzed in combination with bioinformatics tools, served as a basis for the improvement of gene annotation, the elucidation of transcription units (TUs), the identification of putative non-coding RNAs (ncRNAs), and the determination of promoters and ribosome binding sites. This revealed many distinctive properties of the *T. maritima* genome organization relative to other bacteria. This genome has a high number of genes per TU (3.3), a paucity of putative ncRNAs (12), and few TUs with multiple TSSs (3.7%). Quantitative analysis of promoters and ribosome binding sites showed increased sequence conservation relative to other bacteria. The 5′UTRs follow an atypical bimodal length distribution comprised of “Short” 5′UTRs (11–17 nt) and “Common” 5′UTRs (26–32 nt). Transcriptional regulation is limited by a lack of intergenic space for the majority of TUs. Lastly, a high fraction of annotated genes are expressed independent of growth state and a linear correlation of mRNA/protein is observed (Pearson r = 0.63, p<2.2×10^−16^ t-test). These distinctive properties are hypothesized to be a reflection of this organism's hyperthermophilic lifestyle and could yield novel insights into the evolutionary trajectory of microbial life on earth.

## Author Summary

Genomic studies have greatly benefited from the advent of high-throughput technologies and bioinformatics tools. Here, a methodology integrating genome-scale data and bioinformatics tools is developed to characterize the genome organization of the hyperthermophilic, phylogenetically deep-branching bacterium *Thermotoga maritima*. This approach elucidates several features of the genome organization and enables comparative analysis of these features across diverse taxa. Our results suggest that the genome of *T. maritima* is reflective of its hyperthermophilic lifestyle. Ultimately, constraints imposed on the genome have negative impacts on regulatory complexity and phenotypic diversity. Investigating the genome organization of Thermotogae species will help resolve various causal factors contributing to the genome organization such as phylogeny and environment. Applying a similar analysis of the genome organization to numerous taxa will likely provide insights into microbial evolution.

## Introduction

A fundamental step towards obtaining a systems-level understanding of organisms is to obtain an accurate inventory of cellular components and their interconnectivities [1]–[3]. The genome sequence and *in silico* predictions of gene annotation are the starting points for assembling a network. For prokaryotes, these *in silico* approaches detect open reading frames and structural RNAs with varying degrees of accuracy [4]. Recently, multi-omic data generation and analysis studies [5]–[11] have revealed an abundance of genomic features that are not detected computationally such as transcription start sites (TSSs), promoters, untranslated regions (UTRs), non-coding RNAs, ribosome binding sites (RBSs) and transcription termination sites [12]. However, the rate at which multi-omic datasets are being generated is substantially outpacing the development of analysis workflows for these inherently dissimilar data types [13]. Here, multi-omic experimental data is generated and analyzed in conjunction with bioinformatics tools to annotate numerous bacterial genomic features that cannot accurately be detected using *in silico* approaches alone. This methodology was applied to study the genome organization of *Thermotoga maritima*—a phylogenetically deep-branching, hyperthermophilic bacterium with a compact 1.86 Mb genome.

Originally isolated from geothermally heated marine sediment, *T. maritima* grows between 60–90°C with an optimal growth temperature of 80°C [14]. This species belongs to the order *Thermotogales* that have, until recently, been exclusively comprised of thermophilic or hyperthermophilic organisms. Compared to most bacteria, *Thermotogales* are capable of sustaining growth over a remarkably wide range of temperatures. For instance, *Kosmotoga olearia* can be cultivated between 20–80 °C [15]. Recently, the existence of mesophilic *Thermotogales* [16], [17] was confirmed with the description of *Mesotoga prima*, which grows from 20–50 °C with an optimum at 37 °C [18]. Sequencing of *M. prima* revealed that it has the largest genome of all the *Thermotogales* at 2.97 Mb with ∼15% noncoding DNA [19]. *T. maritima*, which grows at the upper-limit known for *Thermotogales*, has one of the smallest genomes in this order and maintains one of the most compact genomes among all sequenced bacterial species (<5% noncoding DNA) [20], [21]. The short intergenic regions in the *T. maritima* genome (5 bp median) resemble those in the genome of *Pelagibacter ubique*, a bacterium that has undergone genome streamlining and has the shortest median intergenic space (3 bp) among free-living bacteria [20]. Although it remains unclear whether *T. maritima* has also undergone streamlining, both organisms encode only a few global regulators (four sigma factors in *T. maritima* versus two in *P. ubique*) and carry just a single rRNA operon. In contrast with *P. ubique*, *T. maritima* displays more metabolic diversity through its ability to ferment numerous mono- and polysaccharides [14], [22].

Thermotogales have been the focus of many evolutionary studies [23]–[25]. Organisms in hydrothermal vent communities, where many Thermotogales have been isolated, are thought to harbor traits of early microorganisms [26]. Phylogenetic analysis of 16S rRNA sequences place the Thermotogae at the base of the bacterial phylogenetic tree [27], [28]; however, Zhaxybayeva et al. [25] determined through analysis of 16S rRNA and ribosomal protein genes that Thermotogae and Aquificales (a hyperthermophilic order) are sister taxa. The authors also determined that the majority of Thermotogae proteins align best with those found in the order Firmicutes [25]; therefore, the exact phylogenetic position of Thermotogae is still unresolved. Nevertheless, members of this phylum are among the deepest branching bacterial species and, as such, prime candidates for evolutionary studies.

Thermophiles such as *T. maritima* implement numerous strategies at both the protein and nucleic acid levels to support growth at high temperatures. For instance, intrinsic protein stabilization is achieved by utilizing more charged residues at the protein surface, encoding for a dense hydrophobic core, and increasing disulfide bond usage [29], [30]. DNA is typically kept from denaturing by introducing positive supercoils via reverse gyrase activity while phosphodiester bond degradation is prevented by stabilization through interaction with cations (e.g. K^+^, Mg^2+^) and polyamines [31], [32]. However, the impact of temperature on genome features essential to gene expression such as promoters and RBSs remains largely unexplored. Bacterial transcription initiation is governed by recognition of promoter sequences by sigma factors, which load the RNA polymerase holoenzyme upstream of the transcription start site (TSS). Translation initiation is predominantly reliant on base pairing between the anti-Shine-Dalgarno sequence found near the 3′-terminus of the 16S rRNA and the Shine-Dalgarno sequence (i.e. the RBS). Therefore, thermophilic macromolecular synthesis machinery must establish and retain contacts with nucleic acids while facing greater thermodynamic challenges.

The integrated approach described here enables an experimentally anchored annotation of several bacterial genomic features including protein-coding genes, functional RNAs, non-coding RNAs, transcription units (TUs), promoters, ribosome binding sites (RBSs) and regulatory sites such as transcription factor (TF) binding sites, 5′ and 3′ untranslated regions (UTRs) and intergenic regions. This is achieved through the simultaneous analysis of genomic, transcriptomic and proteomic experimental datasets with complementary bioinformatics approaches. In addition to providing a valuable resource to the research community, this analysis framework facilitates quantitative and comparative analysis of annotated features across microbial species. For the genome of *T. maritima*, several distinguishing characteristics were identified and their potential causal factors are discussed.

## Results

### An integrative, multi-omic approach for the annotation of the genome organization

**Figure 1:**
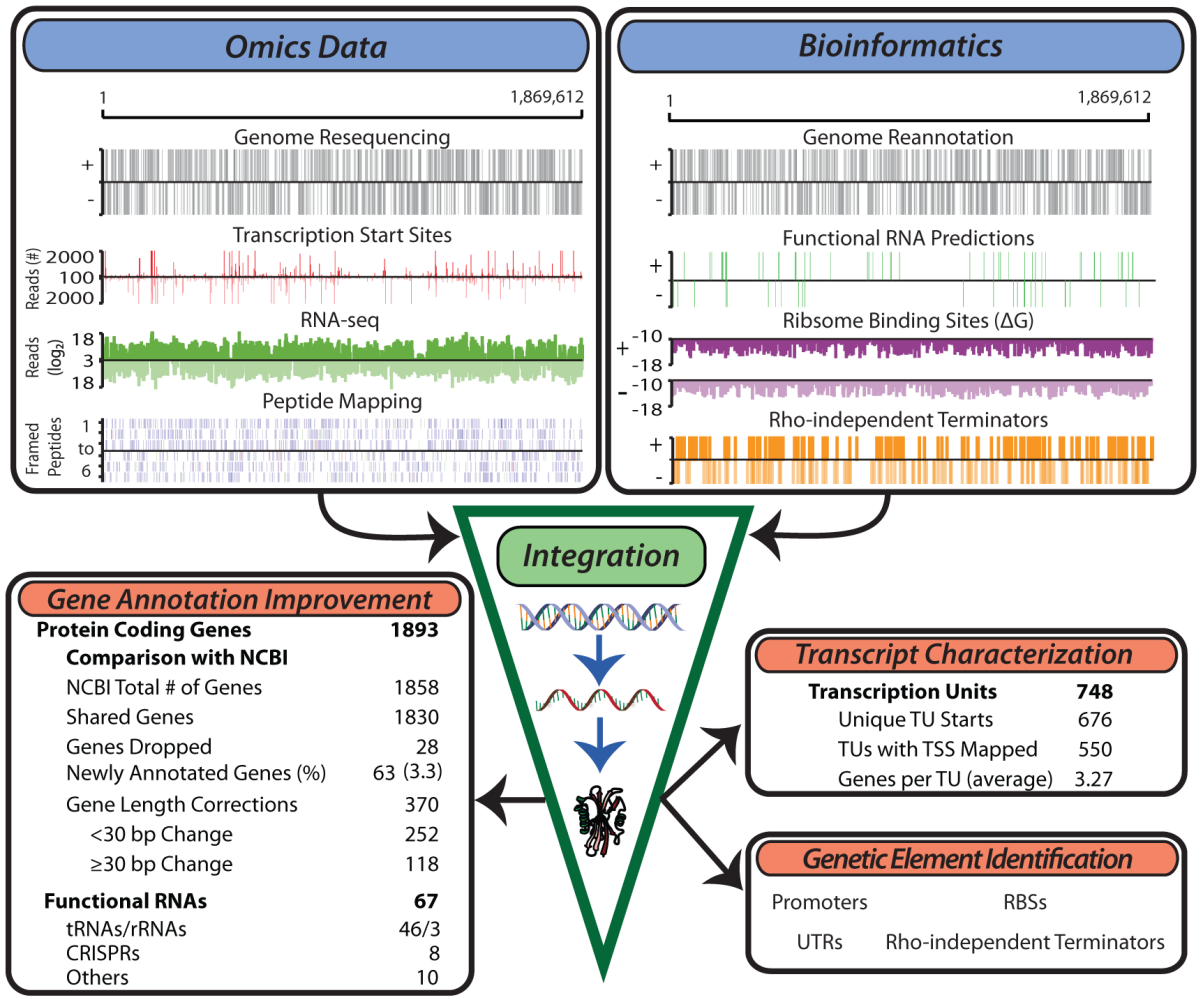
Generation of multiple genome-scale datasets integrated with bioinformatics predictions reveals the genome organization. Experimental data generated for the study of the *T. maritima* genome include genome resequencing, TSS determination, RNA-seq, tiling arrays (not shown) and LC-MS/MS peptide mapping (top left). Bioinformatics approaches used include genome re-annotation, functional RNA prediction, ribosome binding site energy calculations, and determination of intrinsic terminators (top right). Integration of these distinct datasets involves normalization and quantification to genomic coordinates. This experimentally anchors gene annotation improvements, defines the TU architecture, identifies non-coding RNAs and serves as a basis for the identification of additional genetic elements such as promoters and ribosome binding sites.

An integrative workflow was developed to re-annotate the genome of *T. maritima*. The re-annotated genome is the result of the simultaneous reconciliation of multiple omics data sources (Figure 1, upper left) with bioinformatics approaches (Figure 1, upper right). Omics data generated included: (1) genome resequencing, (2) transcription start site (TSS) identification using a modified 5'RACE (Rapid Amplification of cDNA Ends) protocol, (3) transcriptome profiling using both high-density tiling arrays and strand-specific RNA-seq, and (4) LC-MS/MS shotgun proteomics. Transcriptome data were generated from cultures grown in diverse conditions including log phase growth, late exponential phase, heat shock, and growth inhibition by hydrogen (See Materials and Methods). Proteomic datasets include log phase growth and late exponential phase growth conditions. In combination with various bioinformatics approaches, integration of these omics datasets allowed for the definition of gene and transcription units (TU) boundaries with single base-pair resolution. The updated and expanded annotation served as the basis for genome-wide identification of promoters, ribosome binding sites (RBSs), intrinsic transcriptional terminators and UTRs.

#### Annotation of open reading frames (ORFs)

Reannotation of the *T. maritima* MSB8 genome began with whole genome resequencing of the ATCC derived strain. Genome resequencing was prompted by the recent identification of a ∼9 kb chromosomal region in the DSMZ derived strain (DSMZ genomovar, Genbank Accession AGIJ00000000.1) that is not present in the original genome sequence derived from a TIGR strain (TIGR genomovar, Genbank Accession AE000512.1) [33]. Resequencing the ATCC derived strain (presented as the ATCC genomovar, Genbank Accession CP004077) ensured that subsequent analyses referenced an accurate genome sequence. The ATCC genomovar sequence consists of 1,869,612 bp and, like the DSMZ genomovar, carries an ∼9 kb chromosomal region found between TM1847 and TM1848 of the TIGR annotation. The draft genome was annotated using the RAST Pipeline [34] and was then reconciled with the existing TIGR genomovar annotation. The RAST draft annotation had 1,887 protein-coding sequences while the TIGR annotation contained 1,858. Comparison of these two annotations with transcriptome, proteome and bioinformatics datasets resulted in a final annotation containing 1,893 protein-coding sequences (Table S1). The final gene annotation retained a total of 1,830 NCBI annotated genes while 28 NCBI annotated genes were dropped (or replaced) due to a lack of experimental support. An additional 63 genes were annotated based on evidence found in multiple data-types. Furthermore, 370 genes varied in length when comparing the final gene annotation to the NCBI annotation. These discrepancies in gene length were predominantly due to differences in the start codon assignment, thus changing the amino acid sequence at the N-terminus. Gene length annotation differences of less than 10 amino acids were not resolved using the generated datasets without the presence of direct proteomic evidence to support one annotation over the other. However, 118 of these 370 genes (32%) had large discrepancies in their gene length annotation, equaling or exceeding 10 amino acids. For these cases, annotation conflicts were resolved using data from peptide mapping, transcript presence and bioinformatics tools.

#### Annotation of transcription units (TUs)

In addition to the annotation of ORFs, the genome annotation was expanded to include the TU architecture. The TU architecture is defined here to be the genomic coordinates of all RNA molecules in the transcriptome. To expand the annotation to include TUs, transcript bounds were resolved with single base pair resolution using data from RNA-seq and TSS determination. Definition of these bounds was facilitated by bioinformatics approaches; for example, the prediction of intrinsic transcriptional terminators was used to aid in assigning 3′ bounds of transcripts. This approach resulted in the assignment of 748 TUs with a total of 676 unique TSSs (Table S2). The majority of TUs were found to be polycistronic (427, 57%) while the rest of the TUs contain only a single gene (321, 43%). The average TU contains 3.3 genes which is greater than the typical 1–2 genes per transcript observed in other bacteria [7], [35], [36] but similar to those found in archaea [9], [37]. Previous high-resolution studies of microbial transcriptomes have identified the transcription of suboperonic regions as a source of transcriptional complexity [5], [8], [35]. In *T. maritima* 165 TUs (22%) are suboperonic, having their initiation site within a longer TU. This fraction of suboperons observed in *T. maritima* is within the range observed in other bacteria; however, some organisms such as *Helicobacter pylori* have similarly sized genomes (1.67 Mb) but use suboperonic transcription much more frequently (47%, excluding antisense suboperons) [8]. Another source of transcriptional complexity comes from the use of multiple start sites, however, only a small number of *T. maritima* TUs (28, Table S3) were observed to utilize them.

#### Annotation of non-coding RNAs

Beyond facilitating protein-coding gene annotation, transcriptome data provided experimental evidence supporting the bioinformatics prediction of 46 tRNAs, 3 rRNAs, 8 CRISPR cassettes and an additional 10 non-coding RNAs which include riboswitches, leader sequences, RNase P RNA, tmRNA and SRP RNA. These features are included in the final annotation presented here (CP004077, Table S1). Transcription was detected antisense to 19% of annotated genes (Table S4). However, 3′UTRs account for 52% of these antisense transcripts and only 62 antisense transcripts have an experimentally identified TSS. Furthermore, the median log phase FPKM (Fragments Per Kilobase of transcript per Million mapped reads) values are much lower for antisense transcripts (4.5) than those found for protein-coding genes (117). Transcriptome data also enabled identification of 13 putative non-coding RNAs (ncRNAs, Table S5). No secondary structures could be defined for these putative ncRNAs using the prediction algorithms RNAfold [38] and Infernal [39] at 80°C. Four of these putative ncRNAs contain small ORFs (<40 amino acids) but no peptide evidence for these small ORFs was found in the proteomic datasets.

### Identification of promoters and RBSs followed by quantitative intra- and interspecies analysis of binding free energies

**Figure 2:**
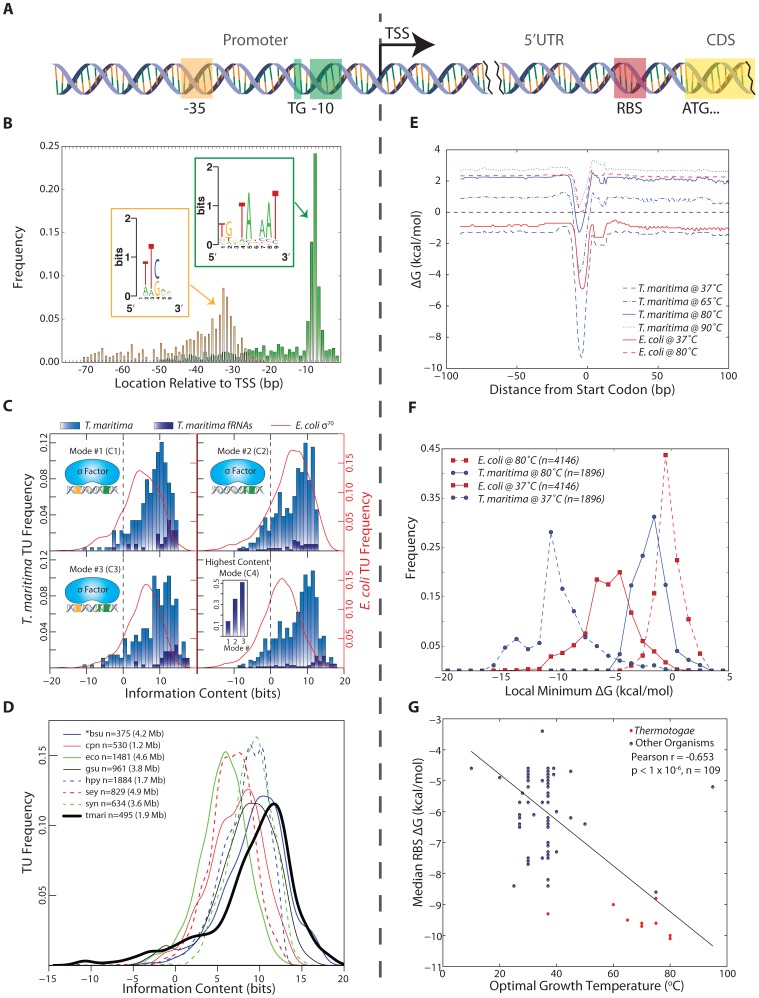
Identification and quantitative comparison of genetic elements for transcription and translation initiation. (A) Schematic showing the position of the promoter upstream of the TSS and the RBS upstream of the translation start codon. (B) The genomic position of the 3′ end of each promoter element is shown relative to the TSS for all *T. maritima* TUs. Promoter elements were identified using a gapped motif search for a −35 hexamer and a −10 nonamer. This revealed an *E. coli* σ^70^ promoter architecture for the housekeeping sigma factor of *T. maritima*, RpoD. The motif for both promoter elements is displayed as a sequence logo (insets). (C) The relative binding free energy of σ^70^ is captured using information content. Each panel shows the distribution of promoter information content for *T. maritima* and *E. coli*. Mode 1 (C1) calculates information content based on σ^70^ contacts with the −35 and −10 hexamer promoter elements (n_tmari_ = 265, n_tmari_fRNA_ = 38, n_eco_ = 650). Mode 2 (C2) represents binding to the extended −10 promoter (n_tmari_ = 676, n_tmari_fRNA_ = 57, n_eco_ = 1,481). Mode 3 (C3) represents σ^70^-binding to both the −35 and the extended −10 promoter elements (n_tmari_ = 274, n_tmari_fRNA_ = 37, n_eco_ = 657). (C4) shows the distribution of information content for all promoters when only the highest scoring mode is considered (n_tmari_ = 676, n_tmari_fRNA_ = 57, n_eco_ = 1,481). The inset shows the highest distribution of functional RNAs across the modes. (D) The σ^70^ binding modes from (C) were used to calculate the promoter information content for seven additional bacterial species. Analogous to (C4), the distribution of information scores when only the highest bit score mode is considered is shown. The organism abbreviations correspond to the following: bsu, *Bacillus subtilis*; cpn, *Chlamydophila pneumoniae* CWL029; eco, *Escherichia coli* K12 MG1655; gsu, *Geobacter sulfurreducens* PCA; hpy, *Helicobacter pylori* 26695; sey, *Salmonella enterica* subsp. enterica serovar Typhimurium SL1344; syn, *Synechocystis* sp. PCC 6803; tmari, *T. maritima* MSB8. The genome size is given in paranthesis. *bsu data is extracted from a highly curated source that is a collection of small-scale experiments and, as such, this distribution is not a genome-scale assessment of promoter strength. (E) The calculated median RBS ΔG for all genes based on the position relative to the start codon. Temperature profiles are shown for *T. maritima* at 37°C (for comparison), 65°C (lower growth limit), 80°C (growth optimum) and 90°C (upper growth limit). Similar profiles are shown for *E. coli* at 37°C (optimal) and 80°C (for comparison). (F) The local minimum RBS ΔG for all genes in a 30 nt window upstream of the annotated start codon generated for *T. maritima* and *E. coli* at 37°C and 80°C. (G) Similar to (F), the median of the local minimum RBS ΔG was calculated and plotted for 109 bacteria against their optimal growth temperature. Species in the Thermotogae phylum (n = 15) are shown in red.

The genome-wide identification of promoter and RBS sites was facilitated by the annotated TU start loci and protein start codons (Figure 2A). Promoter and RBS sequences were then quantitatively analyzed using thermodynamic principles. These same quantitative measures were applied to numerous organisms for interspecies comparison.

#### Annotation-guided search for motifs reveals promoter structures that enable many contacts with RNA polymerase holoenzyme

Bacterial RNA polymerase is recruited predominantly through the binding of sigma factors to promoter regions. A promoter motif search was performed upstream of all unique *T. maritima* TU start sites. This revealed a strongly conserved, *E. coli* σ^70^-like consensus sequence for the housekeeping sigma factor RpoD (Tmari_1457). No motifs were detected for the alternate sigma factors RpoE, SigH and FliA (See Materials and Methods). The RpoD motif has three distinct promoter elements: a −10 hexamer, a −35 hexamer and a 5′TGn element directly upstream of the −10 hexamer (Figure 2B). Individual promoters identified carried combinations of these three elements. The distance between the TSS and the 3′ end of the −10 element was found to be 7 bp (Figure 2B). This is in strong agreement with the expected spacing for the consensus sequence of *E. coli* σ^70^. The same is true of the −35 element though the location of the −35 hexamer is more variable compared with the −10 hexamer partly due to the variability of the spacing between the −10 and −35 promoter elements. Plotting the spacer between the −10 and −35 promoter elements yields a distribution centered around 17 bp, which also is in agreement with the *E. coli* σ^70^ consensus (Figure S1). Furthermore, plotting of genomic AT content upstream and downstream of aligned −10 promoter elements reveals an increase in AT content ∼75 bp upstream of the −10 promoter element (Figure S2). This suggests the presence of UP elements for a subset of *T. maritima* promoters. The α-subunits of RNA polymerase bind to UP elements, facilitating initiation of transcription [40], [41].

#### Quantitative assessment of *T. maritima* promoters indicates high information content across multiple σ^70^ binding modes

The identification of σ^70^ promoter elements enabled the quantitative study of the relative binding free energy associated with individual promoters. The sequence conservation of an individual promoter element (i.e. the information content measured in bits [42]) can be computed through application of molecular information theory and is achieved through quantitative comparison of a given sequence to the average sequence conservation across the genome as measured through the position weight matrix [43] (See Materials and Methods). Information content has been correlated to binding free energy (ΔG) through the second law of thermodynamics [44]–[46], where sequences with high information content are closer to consensus and, therefore, have stronger relative binding free energy (more negative ΔG). Experimental results, both *in vitro* and *in vivo*, have shown that information content is moderately predictive of promoter strength and activity [47].

The information content for individual *T. maritima* promoters was computed using a model of σ^70^ promoters that accounts for the information content of each promoter element and the variation in spacing between the −10 and −35 elements [48]. Using this approach, the information content of each *T. maritima* promoter was determined for three, σ^70^-binding modes that represent the potential contacts between σ^70^ and the promoter elements (Figure 2C1–C3). Plotting the maximum information carrying binding mode for all promoters (Figure 2C4) shows that the vast majority of promoters (90%) have information content greater than zero. This indicates that, for these TUs, σ^70^ binding and transcription initiation is thermodynamically favorable (ΔG<0). Furthermore, the distribution of information content indicates that the median *T. maritima* promoter has 8.7 bits compared to *E. coli* σ^70^ promoters whose median is 5.9 bits. Comparison of *T. maritima* promoters across all modes shows that the extended −10 promoter (−10 hexamer and upstream 5′TGn, Mode 2) provides the highest information for most TUs (63%). Furthermore, an extended −10 promoter combined with a −35 box (Mode 3) yields the highest information content in 25% of all promoters and 51% of functional RNA promoters (Figure 2C4 inset). These RNAs, which are among the most actively transcribed genes, encode promoters with exceptionally high information content (median 12.1 bits).

#### Interspecies comparative analysis reveals that *T. maritima* promoters have high relative sequence conservation

The surprisingly high sequence conservation of *T. maritima* promoters prompted a comparative analysis of information content across multiple bacterial species. The scope of the comparative analysis was limited by the lack of datasets detailing bacterial TSS locations and the association of those TSSs with σ^70^. Publically available datasets for only seven additional, diverse microorganisms met this criteria. The organisms included in the analysis are the Gammaproteobacteria *Escherichia coli* K12 MG1655 [49] and *Salmonella enterica* subsp. enterica serovar Typhimurium SL1344 [50], the Deltaproteobacterium *Geobacter sulfurreducens* PCA [7], the Epsilonproteobacterium *Helicobacter pylori* 26695 [8], the Chlamydiae *Chlamydophila pneumoniae* CWL029 [51], the Cyanobacterium *Synechocystis* sp. PCC 6803 [52] and the Firmicute *Bacillus subtilis* [53]. Since these datasets contain only experimentally confirmed TSS loci, only *T. maritima* TUs with an experimentally confirmed TSS were included in this interspecies comparison (495 TUs out of 676). As before, the information content across all three σ^70^-binding modes was calculated. The distribution of the highest information content mode (Figure 2D) indicates that *T. maritima* promoters are the strongest among all organisms studied, carrying a median of 10.2 bits of information. Thus, among bacteria, *T. maritima* promoter information content associated with σ^70^-binding is relatively high.

#### Analysis of *T. maritima* RBS binding strength reveals strong binding free energies that support translation initiation at 80 °C

The RNA/RNA binding free energy of the Shine-Dalgarno with the anti-Shine-Dalgarno was calculated in a temperature-dependent manner using the gene annotation as a reference point. Across all protein coding genes, the median RBS ΔG was calculated ±100 nucleotides (nt) from the start codon at temperatures ranging from 37 °C to 90 °C (Figure 2E). The position of the lowest ΔG is shown to be 4–6 nt upstream of the start codon, which is in agreement with the optimal RBS location for translation initiation [54]. *T. maritima* is shown to maintain a thermodynamically favorable median ΔG up to its growth temperature maximum of 90 °C [14]. Plotting the distribution of local minimum ΔG's at 80 °C (Figure 2F) reveals that 93% of *T. maritima* protein-coding genes have a RBS with ΔG<0. Calculating RBS free energy distributions at different temperatures (Figure 2F) reveals that at higher temperatures there is a narrowing in the range of observed free energies. *T. maritima* RBSs have a median absolute deviation of 1.30 kcal/mol at 37 °C compared to 0.87 kcal/mol at 80 °C (p = 4.4×10^−33^, Wilcoxon rank-sum test). Comparison of *E. coli* and *T. maritima* RBSs reveals that *T. maritima* RBSs are substantially weaker at their respective optimal growth temperatures (Figure 2F). A large fraction (36%) of *E. coli* genes have a ΔG>0 at 80 °C and would not be capable of supporting hyperthermophilic life. When compared at equal temperatures (Figure 2F, 80 °C) *T. maritima* RBSs are stronger.

#### Interspecies analysis indicates that RBS binding strength is influenced by both optimal growth temperature and phylogeny

To more rigorously test for a relationship between RBS strength and optimal growth temperature, RBS ΔG's were calculated for all genes in 108 additional bacterial species spanning numerous phyla (including 14 members of the Thermotogae phylum). These organisms include psychrophilic, mesophilic, thermophilic and hyperthermophilic microorganisms. A significant linear correlation was found between optimal growth temperature and median RBS ΔG (Pearson r = −0.653, p<1×10^−6^ random permutation test), where increasing optimal growth temperatures trend with a lower median RBS ΔG calculated at 37 °C (Figure 2G). However, the energetic analysis of RBSs applied here is based on the 16S rRNA sequence of the anti-Shine-Dalgarno and, as such, phylogeny is a potential contributing factor to this correlation. To test this, three distance matrices were constructed: (1) for local minimum median RBS ΔG (across all genes in a given genome), (2) for optimal growth temperatures, and (3) for phylogenetic distances determined from 16S rRNA sequences. The Mantel test was then applied to evaluate the correlations among the pairwise distance matrices (Figure S3) allowing for the contribution of optimal growth temperature to be decoupled from phylogeny with respect to RBS strength. This test indicated that both phylogeny and optimal growth temperature impact median RBS strength, with temperature slightly more significant than phylogeny (Mantel Statistic r = 0.37 vs 0.35, p = 1×10^−4^ random permutation test).

### *T. maritima* promoter-containing intergenic regions reveal a unique distribution of 5′UTRs and spatial limitations on regulation

**Figure 3:**
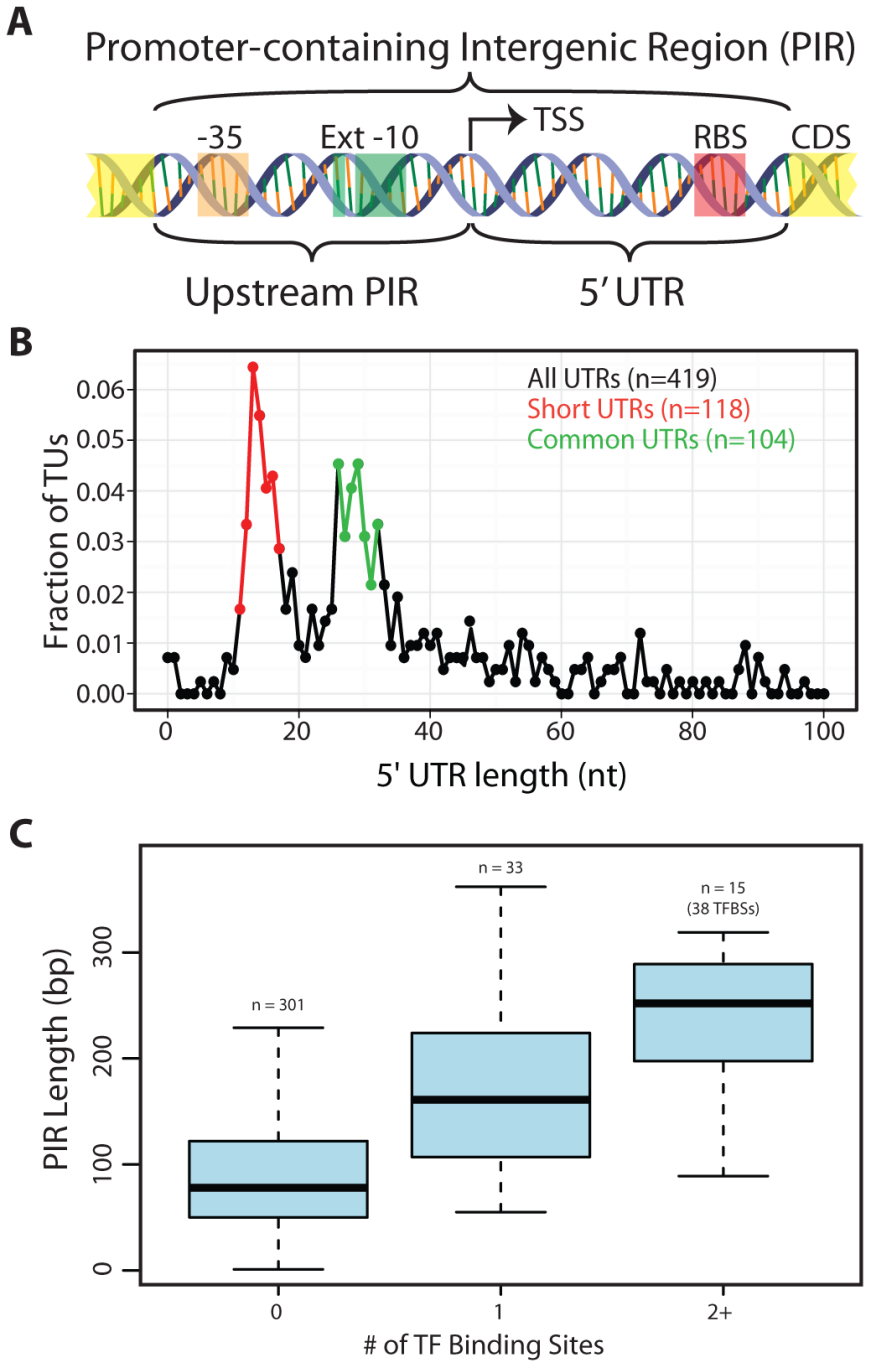
Arrangement of genomic features contained within promoter-containing intergenic regions (PIRs). (A) Schematic of the two subdivisions of the PIR and the genetic elements they typically carry. (B) The 5′UTR distribution is shown for all TUs with an experimentally identified TSS. The Short 5′UTR group (11–17 nt) is shown in red. The Common 5′UTR group (26–32 nt) is shown in green. Transcripts with an annotated functional RNA as the first feature were omitted from the analysis. Though only the first 100 nt are plotted, frequencies are based on the entire set of 5′UTR lengths. (C) A quartile plot of the length distribution of PIRs is shown. PIRs are grouped according to the number of TF binding sites they contain (no TF, a single TF or multiple TFs).

Regulation in *T. maritima* was studied from the vantage point of an organism with extremely short intergenic regions. In both microbes [55] and higher organisms [56] it was shown that the regulatory complexity of an operon positively correlates with the amount of intergenic space found upstream of that operon. Promoter-containing intergenic regions (PIRs) served as well-defined genomic regions for this analysis (Figure 3A). PIRs contain target sites for transcriptional regulation (e.g. promoters and TF binding sites) as well as translational regulation (e.g. RBSs). Each PIR can be divided into two components in relation to the TSS: the sequence downstream of the TSS (the 5′UTR) and the sequence upstream of the TSS.

#### *T. maritima* has a bimodal distribution of 5′UTRs comprised of uncharacteristically “Short” 5′UTRs and “Common” 5′UTRs

*T. maritima* exhibits an unusual bimodal distribution with respect to the length of 5′UTRs (Figure 3B). To date, the 5′UTRs of all other microorganisms follow a unimodal distribution centered at approximately 30 nt [7], [8], [35], [36]. Though *T. maritima* has a distinct peak (local maxima) from 26–32 nt (Common 5′UTR Group), it has a second peak containing shorter 5′UTRs with lengths between 11–17 nts (Short 5′UTR Group). Interestingly, there is underrepresentation of 5′UTRs with lengths between 18–25 nt. Leaderless transcripts were not detected in *T. maritima*, echoing the RNA/RNA binding energy analysis that indicated exclusive use of RBSs for translation initiation.

To better understand the bimodal nature of the 5′UTR distribution, various factors were tested that could differentiate the Short 5′UTR Group from the Common 5′UTR Group and provide insights into the lack of 5′UTRs between 18–25 nt. Factors tested for over- or underrepresentation of the different 5′UTR groups included: (1) gene expression level (both mRNA and protein levels), (2) protein expression normalized to mRNA expression, (3) phylogenetic origin of genes, (4) RBS and promoter strengths, (5) divergent vs. convergent operons, and (6) cellular functional categorization. These factors yielded no discrimination between the Short 5′UTR Group and the Common 5′UTR Group and could not explain the bimodal nature of the 5′UTR length distribution.

#### *T. maritima* PIRs are predominantly too short to permit transcription factor regulation

To enable regulation of transcription, space in the genome must be dedicated to operator sites, which serve as docking locations for TF recruitment. Typically, these sites reside upstream of the TSS, but can also be found downstream of the TSS (in the 5′UTR). An analysis centered on PIRs was chosen to capture the potential for TF binding sites both upstream and downstream of the TSS. A total of 31 TF regulons with a combined total of 91 genomic binding sites were extracted from the RegPrecise database [57]. Mapping of the TF binding sites to the *T. maritima* genome showed that 71 were within PIRs, 12 mapped to intergenic regions not carrying a promoter and the remaining 8 were within or overlapped an annotated gene (Table S6). The length distribution of PIRs without a TF binding site was compared to that of PIRs with TF binding sites (Figure 3C). The median length of PIRs that do not contain a TF binding site is 78 bp. This is significantly shorter than the length of PIRs that carry a single TF binding site (median = 161 bp, Wilcoxon rank-sum test p = 6.9×10^−8^) or multiple TF binding sites (median = 252 bp, Wilcoxon rank-sum test p = 2.8×10^−7^). Thus, the majority of *T. maritima* PIRs do not contain the typical space required to encode a TF binding site.

### *T. maritima* has an actively transcribed genome that is tightly correlated to protein abundances

**Figure 4:**
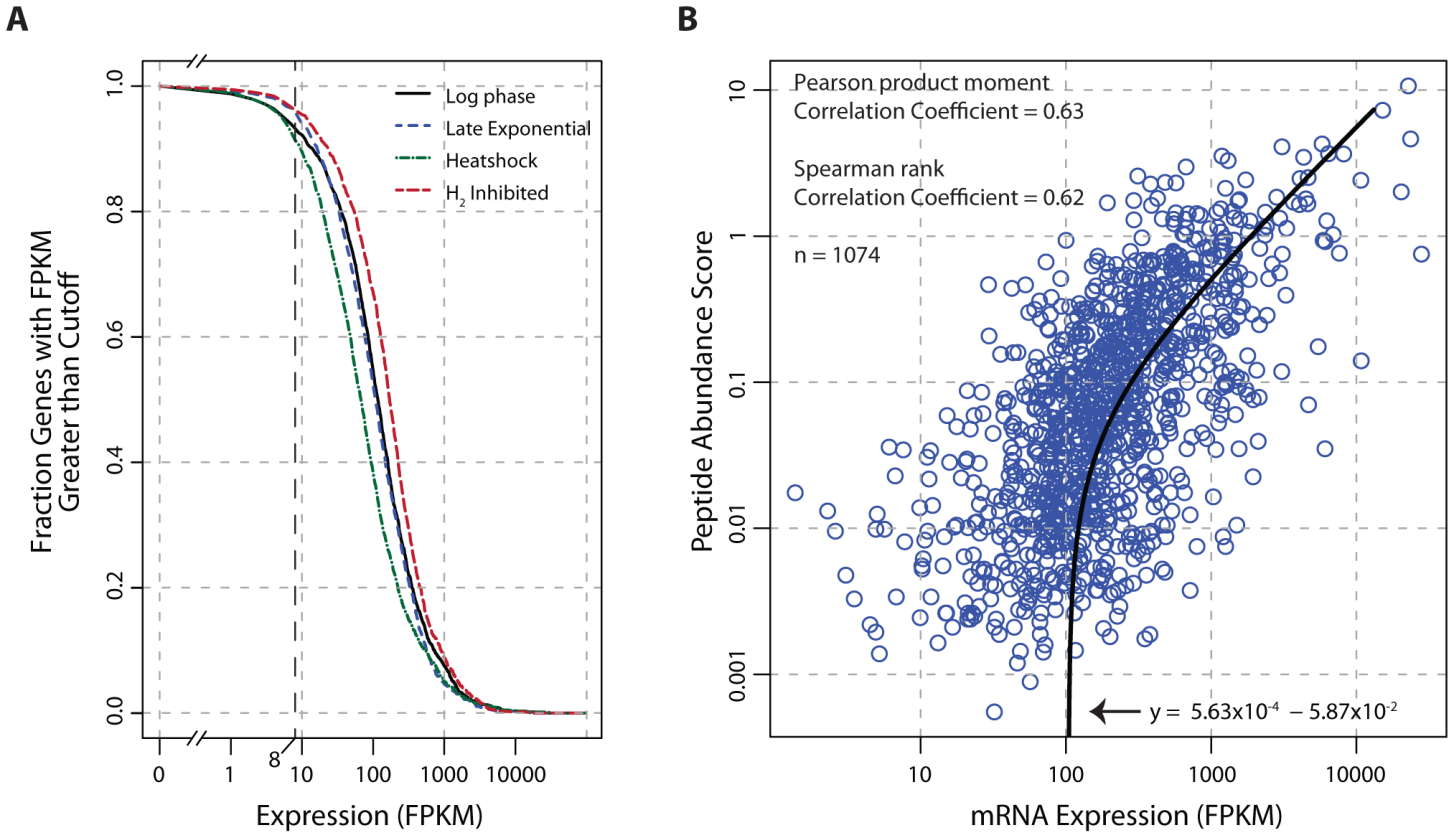
Global analysis of mRNA and protein expression levels. (A) The fraction of transcribed genes as a function of the FPKM threshold. Under growth promoting conditions (log-phase) and early in the transition to stressed conditions (carbon-limited late exponential phase, heat shock, and hydrogen inhibition), 91–96% of the genome is expressed using a conservative FPKM threshold of ≥8. (B) Correlation of mRNA expression and protein abundance. The line of best fit indicates a strong linear relationship (Pearson r = 0.63, p<2.2×10^−16^ t-test) between transcription and translation. The peptide abundance score for each protein was derived by dividing the total spectral count by the number of possible tryptic peptides (400–2000 m/z up to a charge state (z) of 3, hence a maximum fragment mass of 6000). Abbreviations: FPKM, Fragments Per Kilobase of transcript per Million mapped reads; m/z, mass-to-charge ratio.

Transcriptome data indicate that the genome of *T. maritima* is exceptionally active irrespective of growth condition (Figure 4A) with 91–96% of genes expressed above an FPKM threshold of 8. This fraction of genes transcribed is uncharacteristically high compared to other free-living bacteria (see Table S7). Furthermore, translational evidence supporting the high gene expression activity of *T. maritima* is found in the proteomic datasets. In each condition tested, peptide evidence was detected for 74% of the annotated proteins. It is also found that mRNA and protein abundances are tightly linked (Pearson r = 0.63, p<2.2×10^−16^ t-test) (Figure 4B). This correlation is stronger and more significant than those reported in comparable studies for other bacteria [58], [59].

## Discussion

Genome-scale technologies have provided researchers unprecedented access to large volumes of data detailing the composition of a cell. However, approaches for data analysis and interpretation have lagged behind due to the scope and complexity of these data types. Here, we present a framework for multi-omic data analysis that annotates genomic features involved in transcription, translation and regulation. This methodology integrates genome-scale datasets with bioinformatics predictions to produce 1) an improvement of the gene annotation, 2) an experimentally validated TU architecture and 3) the identification of putative antisense, non-coding transcripts and alternative TSSs. Using these annotated genomic features enabled the genome-wide identification of promoters and RBSs, which are difficult to identify solely using *in silico* approaches [60], [61]. Furthermore, the relative binding strength of individual promoters and RBSs was quantitatively measured using thermodynamic principles enabling multi-species comparison of these sequence features. The annotated genome organization served as a scaffold for analyzing regulatory features. Transcription factor regulation was examined with respect to promoter containing intergenic regions while the translational impact of the 5′UTR distribution was considered. The multi-omic data generation and analysis demonstrated here is applicable to many microbial species.

Applying this methodology to study the genome organization of *T. maritima* revealed that it has many distinctive properties compared to other organisms. Genome-scale analysis of promoters showed that *T. maritima* encodes a highly conserved, robust architecture that ensures transcription initiation. Similarly, RBS sequence conservation was shown to be thermodynamically sufficient for translation initiation for almost all *T. maritima* genes at 80°C compared with only a fraction of *E. coli* genes. The distinctive properties of the *T. maritima* genome extend beyond sequence composition and are apparent at the organizational level. The high protein-coding density and minimal intergenic space found in this organism have resulted in a high number of genes per TU, a paucity of putative ncRNAs and few TUs with multiple start sites. Furthermore, transcriptional regulation appears to be limited to a few TUs due to a lack of genomic space in PIRs. Interestingly, the 5′UTR component of the PIR was found to be uncharacteristically bimodal and was comprised of an unusually short grouping of 5′UTRs. Lastly, the constrained genome organization of *T. maritima* is reflected in the physiological state of the cell. Transcription of the vast majority of genes is detected independent of culture condition and the correlation between protein and mRNA is stronger than previously observed in other bacteria.

We hypothesize that the hyperthermophilic lifestyle of *T. maritima* could potentially explain the distinctive characteristics of this organism's genome organization. For instance, the increased sequence conservation of promoter elements and RBSs throughout the *T. maritima* genome may be attributed to the need to maintain gene expression under extreme temperature conditions. Macromolecular interactions (e.g. protein/protein, protein/DNA and RNA/RNA) are intrinsically harder to maintain at higher temperatures. In the case of TF binding sites, it has been shown that each nucleotide deviation from consensus results in a ∼2k_b_T penalty to the maximum binding free energy for a given TF (where k_b_ is Boltzmann's constant and T is temperature) [62]. Increasing the temperature amplifies the binding free energy penalty for every non-conserved base pair. Therefore at 80°C, mismatches between the Shine-Dalgarno and anti-Shine-Dalgarno sequence are especially severe. Thus, *T. maritima* must overcome the intrinsic challenge of recognizing and retaining contact at the initiation site for both transcription and translation. Our data suggests that high sequence conservation of promoter and RBS sequences is one of the mechanisms used by *T. maritima* to ensure sufficient gene expression. This sequence-level adaptation could be analogous to many others observed in thermophilic organisms such as the amino acid composition of proteins [29], [30] and the GC content of structural RNAs [63].

The minimal intergenic space found in the *T. maritima* genome is reminiscent of a streamlined genome, which could explain the limited regulatory capacity observed in this organism. Inflexibility of metabolic regulons has been previously alluded to for other Thermotogales [64]. Here it is demonstrated that, for most TUs, a lack of physical space exists for transcriptional regulation by TFs. Furthermore, the Short 5′UTR group carries the minimum number of nucleotides needed to recruit the ribosome based on Shine-Dalgarno/anti-Shine-Dalgarno interactions [54]. Further reduction in 5′UTR length would abolish translation. Short 5′UTRs also reduce the capacity to regulate by limiting 5′UTR interactions [65], [66].

Though thermodynamics and physical space are hypothesized to contribute to the characteristic features of the *T. maritima* genome, the phylogenetic contribution cannot be dismissed. These potential causal factors are difficult to decouple. For RBSs, we were able to determine the impact of phylogeny and optimal growth temperature on RBS binding strength. By analyzing RBSs from 109 bacterial species spanning many phyla and having a diverse range of optimal growth temperatures we were able to demonstrate that both phylogeny and optimal growth temperature were significant determinants of RBSs sequence composition. However, a recent analysis of genome size among species of the order Thermotogales could not resolve the impact of phylogeny from optimal growth temperature [19]. The authors found that a negative correlation between genome size and optimal growth temperature exists within this order but the correlation did not hold when phylogeny was accounted for in the analysis. Interestingly, this study also found that the number of predicted transcriptional regulators and intergenic space is higher in *Mesotoga prima*, a mesophilic member of the Thermotogales. Thus, the relationship between phylogeny and the genome organization is difficult to elucidate without the generation of more datasets similar to the one presented here.

Thermotogae are an ideal phylum for future investigations on the causal impact of factors such as temperature, intergenic space and phylogeny on genome organization. This phylum contains organisms that are found in many diverse environments with a wide range of optimal growth temperatures. Generating multi-omic datasets and analyzing them using an integrated, quantitative workflow for numerous Thermotogae species would enable assessment of various environmental factors in the context of phylogenetic distance. Furthermore, given their phylogenetic depth, characterization of the Thermotogae will also provide insights in the evolutionary trajectory of microbial life on earth.

## Materials and Methods

### Culture conditions and physiology

*T. maritima* MSB8 ATCC derived cultures were grown at 80°C under anoxic conditions in a chemically defined, minimal medium [67]. Cultures were maintained in either serum bottles or pH-controlled (6.5) fermenters with continuous 80% N_2_, 20% CO_2_ sparging. Maltose and acetate concentrations were measured using an HPLC. HPLC parameters were previously described [68]. The following growth conditions were used for omics analysis: 1) log phase, 2) carbon-limited late exponential phase, 3) heat shock and 4) H_2_ inhibition. Log phase samples were collected from mid-exponential phase cultures grown in 125 mL serum bottles with 50 mL working volume of media and 10 mM maltose as the sole carbon source. Carbon-limited late exponential phase cultures were grown in pH controlled fermenters with pH control and continuous stripping of evolved hydrogen. Cultures were monitored for OD and maltose concentration and samples were collected upon depletion of maltose. The heat shock condition was achieved by rapidly heating mid-exponential phase cultures grown in serum bottles (similar to the log phase condition) to 90°C and sampled after 10 minutes for transcriptome analysis. This has been shown to result in the heat shock response [69]. H_2_ inhibition was achieved by allowing the native evolution of hydrogen to accumulate in serum bottles (similar to the log phase condition). Arrested growth was indicated by successive OD readings that showed no change measured every 30 minutes. Growth profiles for these conditions are shown in Figure S4.

### Genome resequencing and annotation updates

The recent identification of a 9 kb gap in the *T. maritima* MSB8 genome [33] prompted genome resequencing. Genomic DNA was isolated using Promega's Wizard Genomic DNA Purification Kit. Paired-end resequencing libraries were generated following standard Illumina protocols and sequenced on an Illumina GAIIx platform. The updated genome sequence was assembled as follows: (1) Reads were aligned to the 8.9 kb region identified in the *T. maritima* MSB8 DSMZ genomovar (AGIJ00000000.1) [33] and the TIGR genomovar (AE000512.1) sequence using SHOREmap [70] and MosaikAligner (http://bioinformatics.bc.edu/marthlab/Mosaik). (2) Unaligned reads were *de novo* assembled using Velvet [71] to ensure no additional assemblies were present. (3) The sequence was corrected for SNPs and indels detected during read alignment.

An updated genome annotation was generated using the RAST pipeline with the default parameters [34]. Predicted gene sequences were mapped to the AE000512.1 annotation using a bidirectional Smith-Waterman alignment to identify the corresponding locus tags. Instances where ≥30 bp separated the predicted gene length between annotations were reconciled through manual inspection of gene expression data and bioinformatics predictions. Gene length differences <30 bp could not be reconciled (unless peptide data supported only one annotation). In these cases, the updated sequence annotation was retained.

### Transcription start site determination

Total RNA was isolated from log phase cultures using the hot SDS/phenol approach as previously described (http://www.bio.davidson.edu/projects/GCAT/protocols/ecoli/RNApurification.pdf). DNase-treated total RNA samples were recovered using Fisher SurePrep TrueTotal RNA columns. Two biological replicate TSS sequencing libraries were constructed as previously described [7]. Illumina reads were aligned to the updated *T. maritima* genome using the Mosaik Aligner. The number of sequenced reads and the number of aligned reads can be found in Table S10. Only uniquely mapped 5′ ends with ≥5 reads were retained as potential TSSs.

### Transcriptome characterization and gene expression

Tiling array and RNA-seq data were generated under log phase growth, carbon-limiting late exponential phase, heat shock and hydrogen inhibited conditions. Total RNA was isolated using the TRIzol (Invitrogen) extraction procedure followed by DNase treatment and purification using either the Qiagen RNeasy Mini Kit (Tiling Arrays) or the SurePrep TrueTotal RNA columns (RNA-seq).

Custom tiling arrays were synthesized based on the AE000512.1 genome sequence by Roche Nimblegen to carry 71,548 probes with a mean interval of 25 bp. Probe information was remapped to the updated genome sequence. Of the original 71,548 probes, only 125 did not map. Labeled cDNA was generated and processed as previously described [7]. The Transcription Detector algorithm [72] determined probes expressed above background at a FDR = 0.05.

Paired-end, strand-specific RNA-seq was performed using the dUTP method [73] with the following modifications. rRNA was removed with Epicentre's Ribo-Zero rRNA Removal Kit. Subtracted RNA was fragmented for 3 min using Ambion's RNA Fragmentation Reagents. cDNA was generated using Invitrogen's SuperScript III First-Strand Synthesis protocol with random hexamer priming. Illumina reads were aligned to the updated *T. maritima* genome using Bowtie [74] with up to 2 mismatches per read alignment. The number of sequenced reads and the number of aligned reads can be found in Table S10. FPKM values were calculated using Cufflinks [75]. Functional RNA transcripts were excluded from FPKM determination.

### Proteomics, peptide mapping, and protein abundance quantitation

Proteomics samples and data were generally prepared as previously described [76]. In summary, triplicate samples of both log phase and late exponential phase culture were lysed by French press, and proteins were extracted into global, soluble, and insoluble fractions. The three protein fractions were digested with trypsin (Promega) for 4 h at 37°C and then cleaned-up using C18 or SCX SPE columns (Supelco), as appropriate. Resulting peptide samples were separated in the first dimension by high pH HPLC (Agilent) and then analyzed by LC-MS/MS using C18 resin (Phenomenex) with an expontial gradient on a custom built LC platform coupled to a linear ion trap (LTQ) or a Velos Orbitrap mass spectrometer (Thermo Scientific) operated in data dependent mode. Peptides were identified by SEQUEST (Thermo Scientific) against a six-frame translation of the *T. maritima* genome with no protease specified in the search. Xcorr values were refined to conform to generally accepted criteria and were applied to result in a false discovery rate of 0.16% at the peptide level. Non-quantitative peptide-level data can be found in Table S8.

Normalized protein abundances can be found in Table S9. Quantitative Peptide-level data was extracted from Lerman et al. [77] and mapped to the CP004077 genome annotation. The following criteria were used to filter proteins for quantitative analysis: 1) the protein has a total spectral count ≥2 across all conditions (minimum of two unique peptides or a single unique peptide with two observations), 2) the protein has ≥1 observed peptide under log phase since our data was correlated against log phase transcriptome data. Redundant peptides (i.e. peptides mapping to multiple protein entries) were excluded from the analysis to minimize potential ambiguity. For quantitative analysis, we normalized the observed spectral counts for each ORF by the number of possible fully tryptic peptides in the ORF. The number of possible fully tryptic peptides for each ORF was determined using the Protein Digestion Simulator (http://omics.pnl.gov/software/ProteinDigestionSimulator.php). Default settings were used, except the parameter “Max Missed Cleavages” was set to 0 and “Minimum Residue Count” was set to 6. These options require fully tryptic peptides of at least length 6. This program only considers peptides 400–2000 m/z up to a charge state (z) of 3, hence a maximum fragment mass of 6000.

### Promoter element motif analysis and position weight matrix (PWM) generation

The process of determining individual σ^70^ promoter elements upstream of each unique TU start in *T. maritima* was an iterative process, involving two software packages: BioProspector [78] and MEME [79]. BioProspector is able to identify gapped motif elements so it was used to initially identify *T. maritima* motifs. In BioProspector, sequences 75 bp upstream of TU starts were searched for bipartite elements (6 and 9 bp in width) with a 10–25 bp allowable gap and visualized through WebLogo [80]. MEME provides deterministic position-weight matrices appropriate for information content calculations. The −10 and extended −10 boxes were searched [−1 to −18] upstream of the TSS while the −35 box was searched [−20 to −44]. *E. coli* TUs annotated with σ^70^ promoters and experimentally validated TSSs in the EcoCyc Database (version 15.0) [49] were extracted for comparative analysis.

A similar approach was applied to identify promoter motifs for alternative sigma factors. *T. maritima* has three annotated alternative sigma factors: RpoE (Tmari_1606), SigH (Tmari_0531) and FliA (Tmari_0904). For RpoE and SigH, the upstream region of TUs having genes showing high differential expression under a given stress condition (heat shock, hydrogen inhibited and carbon-limited late exponential phase) were searched for motif elements. The upstream regions of flagellar gene encoding TUs were searched for a FliA motif. However, no sequence motif could be detected for any of the three alternate sigma factors.

### Information content calculations

Position weight matrices (PWMs) for each promoter element were converted to individual information weight matrices using the following formula established in the field of molecular information theory [43]: R*_iw_*(*b*, *i*) = 2−(−log_2_f(*b*, *i*)), where f(*b*, *i*) is taken to be the probability of observing base *b* at position *i*. The individual information of a sequence, I*_seq_*, was calculated by summing the relevant entries of R*_iw_*. For any particular sequence, only one entry of R*_iw_* is relevant among 4 bases for each position *i* in the sequence. I*_seq_* is measured throughout in bits since the log was base 2 in converting the PWM to R*_iw_*.

I*_seq_* reflects sequence conservation for a single sequence, but natural promoters are often formed by multiple promoter elements, each with their own sequences and corresponding I*_seq_* values. When multiple elements are present, variable length spacers are frequently found between the elements. We applied an approach previously described by Shultzaberger et al. [48] to properly account for all possible promoter elements and the variation in their spacing. This allowed us to assess total sequence conservation for an entire promoter. For each promoter, the information content for a particular binding mode was calculated based on the formulas: (1) Mode 1: I*_seq_whole_promoter_* = I*_seq_*(−10 element)+I*_seq_*(−35 element)−GS(*d*); (2) Mode 2: I*_seq_whole_promoter_* = I*_seq_*(extended−10 element); (3) Mode 3: I*_seq_whole_promoter_* = I*_seq_*(extended−10 element)+I*_seq_*(−35 element)−GS(*d*). GS(*d*) is ‘gap surprisal’ accounting for variable spacing (of length *d*) between the −10 and −35 elements. GS(*d*) penalizes for unexpected spacing given the major groove accessibility of B-form DNA and was defined as in equation (3) in Shultzaberger [48] with no small-sample correction factor as the analysis here is performed at genome scale. In accordance with the Shultzaberger model, the space between the −10 and −35 elements was restricted to 15–20 bp as measured from the 3′ end of the −35 element and the 5′ end of the −10 element. This limit on the spacer distance I*_seq_whole_promoter_* is measured in bits.

### Ribosome binding site energy calculations

The anti-RBS sequence 5′-UCACCUCCUU-3′ (3′ end of the 16S rRNA) was selected for this study. The hybrid-2s program in the UNAFold software package [81] was used to compute hybridization energies (ΔG) for all possible 10-mers over the temperature range 20–100°C. This dictionary was mined for three applications: (1) binding energy values for all 10-mer sequences in the updated *T. maritima* genome were computed to aid in annotation improvement, (2) the median positional ΔG for all CDSs ±100 bp from the start codon, and (3) the local minimum ΔG for all CDSs 30 bp upstream of the start codon. RBS binding energies across 109 organisms were calculated using this dictionary. Optimal growth temperatures for all non-Thermotogae bacteria were collected from Takemoto et al. [82] and the protein coding gene annotation for each bacterium was extracted from NCBI. CDS data for all Thermotogae with a complete genome sequence were extracted from NCBI with the exception of *T. maritima* for which the annotation generated in this study was used. For each organism, the median RBS ΔG was calculated from the set of minimum RBS ΔG's found for each CDS 30 bp upstream of the annotated start codon. Three distance matrices were constructed for analysis of the 109 bacterial species for which optimum growth temperatures were found. The matrices included are as follows: (1) the absolute difference of median RBS strength values, (2) the absolute difference of optimal growth temperatures and (3) the distance matrix generated by aligning full-length 16S rRNA gene sequences using ClustalW2 (slow mode) followed by the phylogenetic tree generation script (http://www.ebi.ac.uk/Tools/phylogeny/) with default settings. Next, the Mantel test, which tests the correlation between two distance matrices, was applied to compute the significance of various correlations. The ‘vegan’ package of R was used with its default settings.

### Rho-independent terminator site determination

Intrinsic terminators were predicted using the TransTermHP program [83]. To avoid bias introduced by annotation, no genome annotation was used in prediction of Rho-independent terminators. Only terminator structures predicted with a “100%” confidence score were included in the curation of TUs.

### Prediction of small RNAs

Small RNAs were predicted with Infernal [39] using cmsearch with default settings against the Rfam 10.0 Database [84] of small RNA families. sRNAs with an E-value<0.01 were manually curated to verify expression. These sRNAs were checked against the sRNA predictions from Rfam and fRNA-DB (http://www.ncrna.org) based on the AE000512.1 genome sequence.

### Transcription unit assembly

TU assembly was accomplished through an iterative procedure beginning with tiling array expression data. Tiling array data was processed with two Bioconductor packages for transcript segmentation based on change point analysis: tilingArray (http://www.bioconductor.org/packages/2.2/bioc/html/tilingArray.html) and DNAcopy (http://www.bioconductor.org/packages/2.3/bioc/html/DNAcopy.html). Manual comparison of the output from both packages with array data was used to refine the automated set of transcriptional segments. Additional datasets and bioinformatics predictions were added and manually curated to fully characterize the TU assembly. TSS and RNA-seq data provided single-base pair resolution of segment boundaries. Intrinsic terminator predictions were also used for 3′ boundary definition. ncRNAs were identified using the transcript segments. Transcribed regions not associated with a TU and with length exceeding 68 nt (the combined length of the paired end reads with no insert separating them) were quantified using Cufflinks to generate FPKM values across all RNA-seq conditions. Regions with at least two conditions showing FPKM values >8 were retained as putative ncRNAs.

### Transcription factor binding site mapping

TF binding sites were extracted from RegPrecise [57] and coordinates were mapped to the updated genome. Table S6 has the TF binding sites used in Figure 3C.

### Data deposition

The *T. maritima* MSB8 ATCC (genomovar) genome and annotation are found under Genbank Accession CP004077. RNA-seq, TSS, and tiling array datasets are available in the Gene Expression Omnibus under Accession GSE37483. Proteogenomic data are made available through PNNL (http://omics.pnl.gov) and in Table S8.

## Supporting Information

Figure S1 Spacing between the −10 and −35 promoter elements. The distribution of the number of base pairs separating the −10 promoter element from the −35 promoter element for each unique transcription start site.
(PDF)

Figure S2 AT content in the regions surrounding promoters. The AT fraction is shown for each promoter motif determined. The plot is shown ± 300 bp with respect to the 3′ end of the −10 promoter element.
(PDF)

Figure S3 Mantel test statistic r for comparison of distance matrices. Three distance matrices were constructed: (1) absolute difference of median RBS strength values (this matrix is denoted R), (2) absolute difference of optimal growth temperatures (this matrix is denoted T), and (3) a distance matrix generated by aligning full-length 16S rRNA gene sequences (this matrix is denoted P). The rows and columns of these matrices are the organisms for which optimal growth temperature was available. The Mantel test, which tests the correlation between two distance matrices (denoted (X,Y)), was applied to compute the significance of various correlations. The ‘vegan’ package of R was used with its default settings. The test statistic r falls in the range [−1 to +1], where −1 indicates strong negative correlation and +1 indicates strong positive correlation. An r value of 0 indicates no correlation. Finally, Partial Mantel test statistics were computed using all three distance matrices. In each of these tests, a partial correlation conditioned on the third matrix (denoted (X,Y |Z)) was computed. In all Mantel tests, the results using the Pearson method are reported. All tests had significant p-values (p<0.001).
(PDF)

Figure S4 Growth physiology and sample points for omics data. (A) A typical batch growth experiment is shown in serum bottles. *T. maritima* was grown on maltose minimal media in 125 mL serum bottles with 50 mL working volume. Optical density and hydrogen accumulation (as measured in the headspace) is shown. Arrow 1 marks the sample point for the log phase condition and for conducting heat shock. Arrow 2 marks the sample point for H_2_ inhibited growth. (B) A typical batch growth profile using a pH controlled bioreactor with continuous H_2_ removal by sparging 80% N_2_, 20% CO_2_. Optical density, maltose concentration, acetate concentration and pH profiles are shown. Arrow 3 marks the sample point for carbon-limited late exponential phase.
(PDF)

Table S1 Updated *T. maritima* genome annotation.
(XLSX)

Table S2 *T. maritima* transcription unit assembly.
(XLSX)

Table S3 Potential Alternative start sites.
(XLSX)

Table S4 Detected antisense transcripts.
(XLSX)

Table S5 Putative ncRNAs.
(XLSX)

Table S6 Transcription factor binding sites mapped to the new genome sequence.
(XLSX)

Table S7 Fraction of the genome detected in free-living microorganisms compared with *T. maritima*.
(XLSX)

Table S8 Non-quantitative peptide-level data mapped to the updated *T. maritima* annotation.
(XLSX)

Table S9 Normalized protein abundance data.
(XLSX)

Table S10 Sequencing statistics for transcriptome datasets.
(XLSX)
