# Supplementary figures and images for: The Genome Organization of Thermotoga maritima Reflects Its Lifestyle
Source: PLoS Genet. 2013 Apr 25;9(4):e1003485. doi: 10.1371/journal.pgen.1003485 (PMC3636130; doi:10.1371/journal.pgen.1003485)

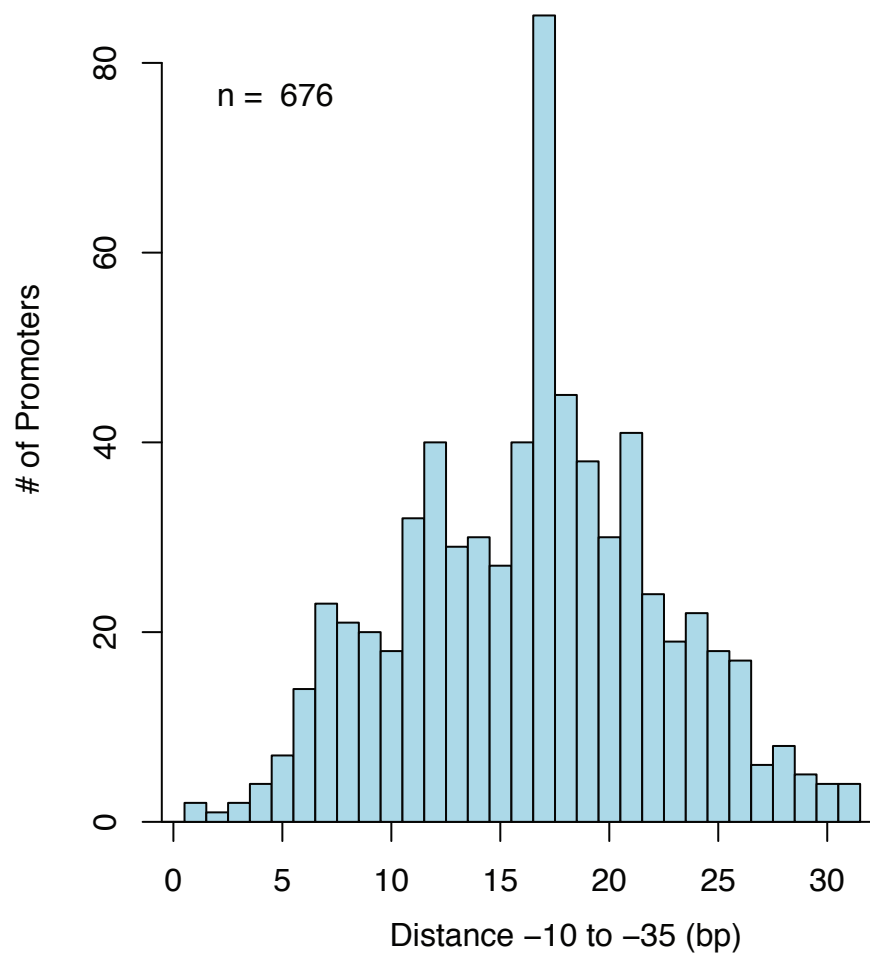

Figure S1

Supplement: Figure S1 — Spacing between the −10 and −35 promoter elements. The distribution of the number of base pairs separating the −10 promoter element from the −35 promoter element for each unique transcription start site. (PDF) [file pgen.1003485.s001.pdf]

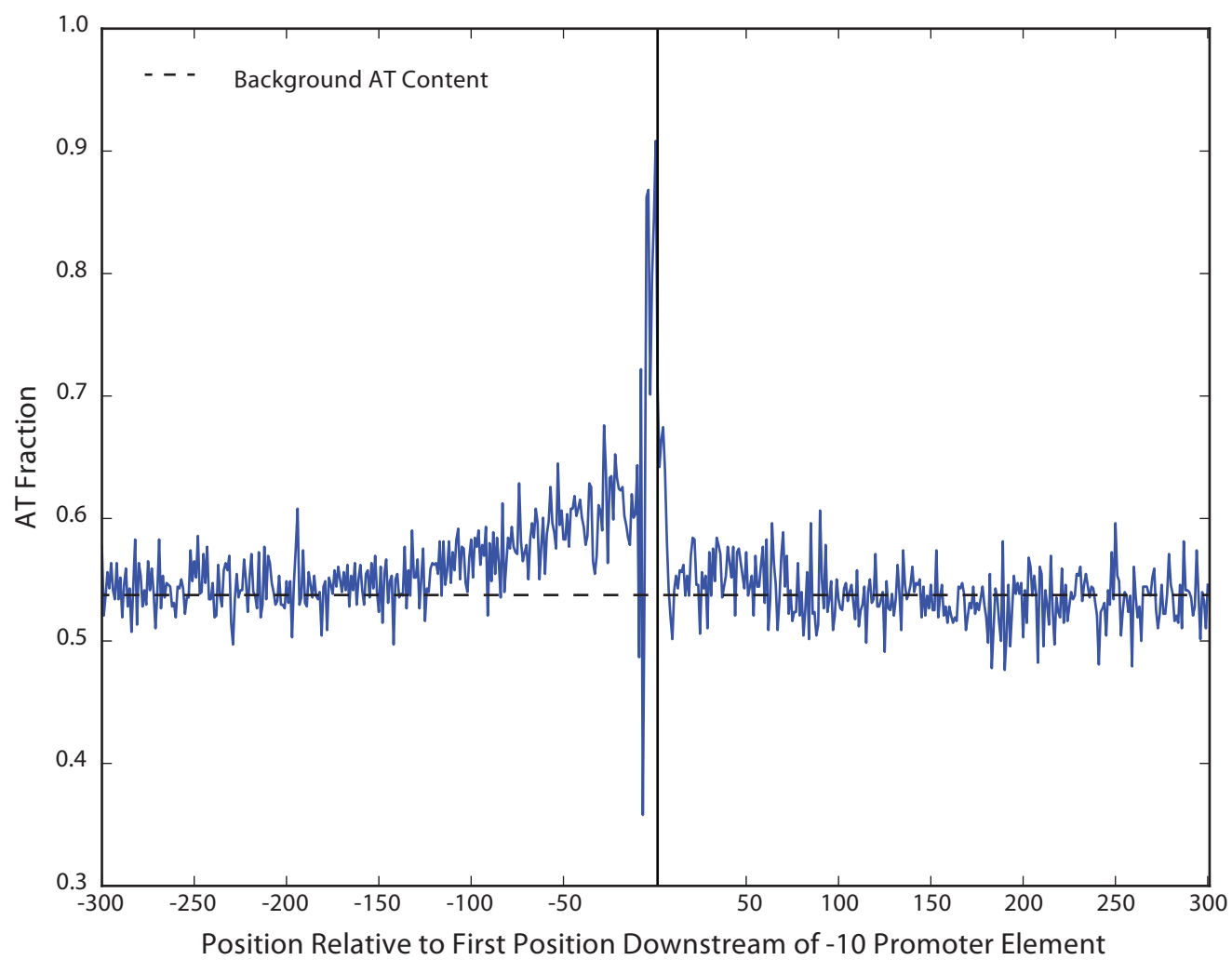

Figure S2

Supplement: Figure S2 — AT content in the regions surrounding promoters. The AT fraction is shown for each promoter motif determined. The plot is shown ± 300 bp with respect to the 3′ end of the −10 promoter element. (PDF) [file pgen.1003485.s002.pdf]

**A**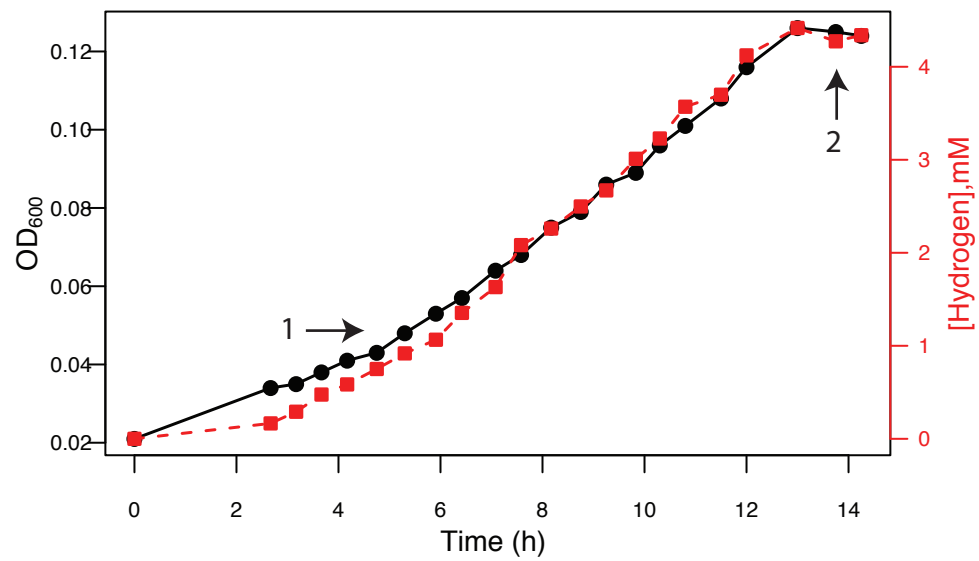**B**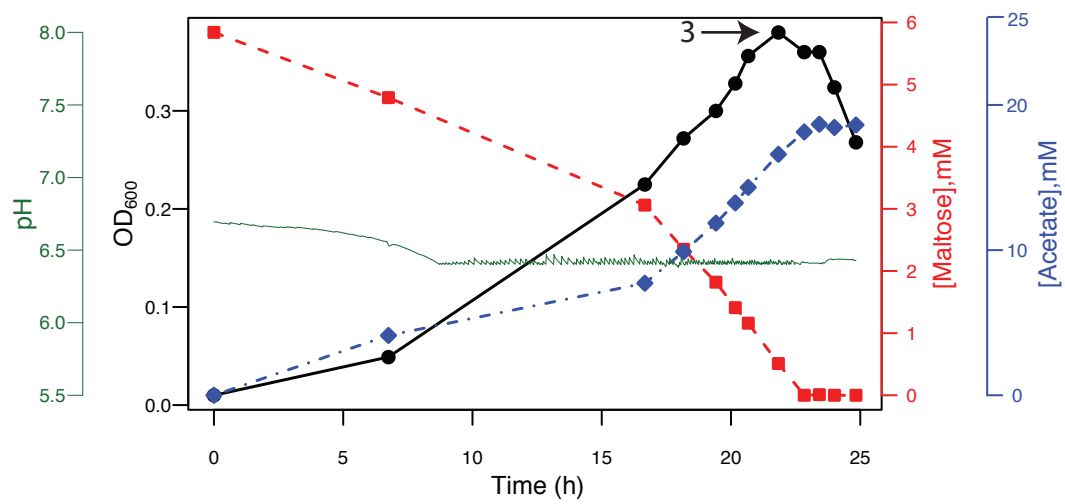**Figure S4**

Supplement: Figure S4 — Growth physiology and sample points for omics data. (A) A typical batch growth experiment is shown in serum bottles. T. maritima was grown on maltose minimal media in 125 mL serum bottles with 50 mL working volume. Optical density and hydrogen accumulation (as measured in the headspace) is shown. Arrow 1 marks the sample point for the log phase condition and for conducting heat shock. Arrow 2 marks the sample point for H2 inhibited growth. (B) A typical batch growth profile using a pH controlled bioreactor with continuous H2 removal by sparging 80% N2, 20% CO2. Optical density, maltose concentration, acetate concentration and pH profiles are shown. Arrow 3 marks the sample point for carbon-limited late exponential phase. (PDF) [file pgen.1003485.s004.pdf]
