# Supplementary material for: The Genome Organization of Thermotoga maritima Reflects Its Lifestyle
Source: PLoS Genet. 2013 Apr 25;9(4):e1003485. doi: 10.1371/journal.pgen.1003485 (PMC3636130; doi:10.1371/journal.pgen.1003485)
Supplement: Figure S3 — Mantel test statistic r for comparison of distance matrices. Three distance matrices were constructed: (1) absolute difference of median RBS strength values (this matrix is denoted R), (2) absolute difference of optimal growth temperatures (this matrix is denoted T), and (3) a distance matrix generated by aligning full-length 16S rRNA gene sequences (this matrix is denoted P). The rows and columns of these matrices are the organisms for which optimal growth temperature was available. The Mantel test, which tests the correlation between two distance matrices (denoted (X,Y)), was applied to compute the significance of various correlations. The ‘vegan’ package of R was used with its default settings. The test statistic r falls in the range [−1 to +1], where −1 indicates strong negative correlation and +1 indicates strong positive correlation. An r value of 0 indicates no correlation. Finally, Partial Mantel test statistics were computed using all three distance matrices. In each of these tests, a partial correlation conditioned on the third matrix (denoted (X,Y |Z)) was computed. In all Mantel tests, the results using the Pearson method are reported. All tests had significant p-values (p<0.001). (PDF) [file pgen.1003485.s003.pdf]

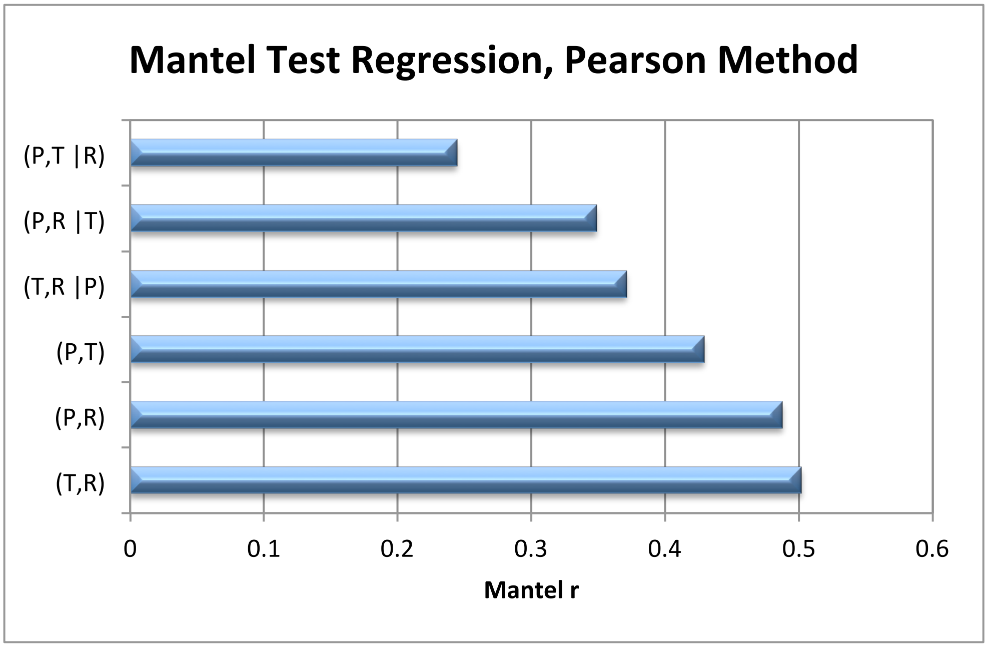

Abbreviation	Test Comparisons	Mantel Statistic r	p-value
(T,R)	Temp vs RBS	0.50	1.00E-04
(P,R)	Phylogeny vs RBS	0.49	1.00E-04
(P,T)	Phylogeny vs Temp	0.43	1.00E-04
(T,R |P)	Temp vs RBS, Phylogeny Corrected	0.37	1.00E-04
(P,R |T)	Phylogeny vs RBS, Temp Corrected	0.35	1.00E-04
(P,T |R)	Phylogeny vs Temp, RBS Corrected	0.24	1.00E-04

Figure S3
